# Road Safety Perception Questionnaire (RSPQ) in Latin America: A Development and Validation Study

**DOI:** 10.3390/ijerph18052433

**Published:** 2021-03-02

**Authors:** Fabricio Esteban Espinoza Molina, Blanca del Valle Arenas Ramirez, Francisco Aparicio Izquierdo, Diana Carolina Zúñiga Ortega

**Affiliations:** 1 Institute of Automobile Research Francisco Aparicio Izquierdo (INSIA), Universidad Politécnica de Madrid (UPM), 28031 Madrid, Spain; blanca.arenas@upm.es (B.d.V.A.R.); francisco.aparicio@upm.es (F.A.I.); 2 Transportation Engineering Research Group, Universidad Politécnica Salesiana, 010105 Cuenca, Ecuador; dzuniga@ups.edu.ec

**Keywords:** road safety, road safety perception, questionnaire validation, drivers’ behaviors

## Abstract

*Background*: Although public bodies need to know drivers’ perception of road safety, in Latin America there are no valid and reliable instruments that propose an integral dimensionality. The objective of this study was to design and validate a Road Safety Perception Questionnaire (RSPQ). *Methodology*: The design included a review of the available evidence and expert knowledge to select the dimensional items for the instrument. A pilot test was carried out to determine possible corrections and adjustments to the questionnaire, after which a Confirmatory Factor Analysis was performed on a stratified sample of 736 Ecuadorian drivers to determine its reliability and construct validity. *Results*: The results suggest that the RSPQ has a clear factorial structure with high factorial weight items and good internal consistency. The results of the 41-item model grouped into six dimensions (human, vehicle, road infrastructure, regulatory framework and intervention measures, socioeconomic and driving precautions) obtained the best adjustment indexes at the absolute, incremental and parsimonious levels. *Conclusions*: The preliminary RSPQ evidence can be considered a valid and reliable instrument to assess drivers’ perception of road safety.

## 1. Introduction

Road safety is a high priority issue in all countries, especially in Latin America. The WHO Global Status Report has reported a high annual number of road traffic deaths (approximately 1.35 million [1]), while the situation analysis indicates that most casualties are due to human-related issues so that their handling has become the highest dynamic target of road safety actions [2].

Traffic accidents are a national and social problem due to their high cost in material damages and human lives [3], particularly in certain regions such as South America, where there were 154,997 deaths due to traffic accidents in 2016, i.e., 11% of the total deaths for this reason worldwide. The traffic accident mortality rate in all South America per 100,000 inhabitants is 15.6, 20.9 in the Andean Region and 18.4 in the Southern Cone, with higher numbers in countries with medium incomes than in those with higher incomes [4]. Ecuador is no exception to this rule and 2180 people died there for this reason in 2019, the highest number since 2015, when 2138 people were killed. Of the total number of traffic accidents recorded by the National Traffic Agency (ANT), 96.08% were due to human factors such as distraction, negligence, and in some cases a lack of knowledge of the highway code [5]. This indicates a difficult situation and significantly reducing the number of road accident deaths is a complex task.

These accidents can be attributed to various causes, including driving conditions, bad weather, drivers’ behavior and experience [6], and excessive speed [6,7]. Drivers’ behavior is also known to be influenced by the road infrastructure and the physical and social conditions [8]. The road design and safety characteristics are known to influence vehicle speeds [9,10,11,12]. Distraction, lack of attention [13], health problems, reckless driving, and breaking driving rules are the main factors in the rise of the number of traffic accidents [14], while they can be avoided by keeping a vehicle in good conditions, training and education in safe driving [15,16]. There are also significant differences in the driving practices between countries [17] and drivers’ conduct varies significantly between different countries, with different perceptions of driving risks [18]. Therefore, there are many variables influencing road accidents that need to be studied.

A good departure point is to have a road accident information system, which could be not reliable, incomplete, and inaccessible, making it impossible to carry out in-depth research studies. Other resources are questionnaires which could allow knowing the habits, perceptions, and beliefs of road users.

As part of this shortcoming, no surveys have been conducted to assess the behavior of road users (drivers, pedestrians, cyclists) with recognized and validated instruments. Ecuador is not an exception and needs further research in this area.

The road safety perception questionnaire to be designed takes into account these singularities of the variables causing road accidents described above.

In this sense, it takes considerable time for countries to create a national database of information on road traffic crashes, the implementation of which depends on government policies. Having such information is important for decision making and one of the data sources for assessing road users’ perception of road safety is the survey, which is an important and relatively inexpensive source. Considering the efforts to improve road safety [1], there is an important need to know the perception of road safety of individuals [19] (drivers, pedestrians, cyclists, etc.).

Traffic psychology essentially deals with road safety, emphasizing the study of the human factor as the main cause of risks [20], while drivers’ behavior and experience are fundamental factors involved in safety [21] and contribute valid components to systematically understanding the problem. However, this is only one of the elements involved, since a wider perspective can identify different dimensions and consequences that should be investigated [22,23] such as social interaction and social values [24].

The driver’s behaviour, his or her assessments, and beliefs, as well as his or her physical and psychological conditions, influence the way he or she behaves in such a complex environment as driving. Certain psychophysical conditions are fairly well known, which greatly increase the risk involved in the activity of driving, but we still have a lot to understand how drivers perceive risk in the face of other road safety factors (vehicle, regulations, road infrastructure), which are at the root of particularly risky behaviour, so that local reference frameworks can to be constructed to explain the behavior and characteristics of all the agents involved in road safety [25], while the socio-cognitive factors can help understand the underlying motives for certain types of conduct [26,27,28]. It is therefore urgent to design integral and valid instruments to measure the dimensions of road safety in order to analyze the drivers’ perceptions and thus support the decision makers in road safety policies.

Attitudes to road safety have been measured by questionnaires, interviews, pyscho-physiological registers, and observation tests [29]. Of course, there have been many attempts to observe and understand drivers’ behavior in different situations by various methods, including virtual reality laboratory tests, localization data, video recordings based in vehicles, and direct observation [19]. However, it has been suggested that these methods give results that cannot be generalized outside the context in which they were obtained [30], so that self-informing questionnaires, due to their low cost and rapidity, are more often used than the above-mentioned instruments and most researchers opt for this type of measurement.

On the other hand, socio-cognitive factors, like attitudes and beliefs can help to understand the underlying motives of unsafe driving behavior [31] and various countries include these dimensions in following road safety. For example, the Social Attitudes to Road Traffic Risk in Europe (SARTRE) report [32,33] aimed to study perceptions and conduct of drivers in various countries to driving risks and to assess their evolution and the possible influence of actions in different road safety areas. Another survey studied the social and cultural dimensions of drivers’ behavior in the USA [34] to identify and evaluate key indicators of drivers’ attitudes and behavior since 2008 was called the Traffic Safety Culture Index, and included dimensions such as distracted driving, aggressive driving behaviors, drowsy driving, and impaired driving [35], but did not include vehicle-related factors, infrastructures and factors such as e.g., their age, safety systems, maintenance, signaling and highway lighting, among others.

Various instruments have been created to assess drivers’ habits [36] as regards human conduct and road regulations [37], or attitudes [38,39], although they have not yet been widely applied in South America. The Mexican State Mobility Survey was used to determine the population’s perception of mobility, road safety and highway regulations [40]. Trógolo [41] developed a short self-sufficiency scale in Argentina for measuring drivers’ self-evaluation and their degree of confidence when driving in different situations. As far as we know there have been no valid integral surveys on road safety in South America that include weather and driving conditions [25] and little scientific research has been done on this subject in Ecuador [42], largely due to a lack of valid methods, and instruments. Muñiz has pointed out that these instruments should be specially constructed and validated for different cultural contexts so that the data collected can be generalized and validated as a reliable guide for decision-makers [43]. As far as we know, no comparable and reliable data has been produced on drivers’ behavior in Ecuador.

In this work, we present the design and validation of a questionnaire focused on the five road safety factors and related indicators, being the main ones: (a) Human (FH), (b) Vehicle (FV), (c) Road infrastructure (FIV), (d) Regulatory framework and intervention measures (FN) and (e) Socio-economic (FS), which are interrelated, forming the so-called MIICA model [44].

The Road Safety Perception Questionnaire takes into account some particularities such as culture and level of development. Furthermore, it is a tool specially designed for countries that have high underreporting and do not collect all the variables necessary for an in-depth analysis of road crashes, as is the case of many in South America. An on-going work dealing with data analysis of the application of the survey to a wide sample of local drivers has two main objectives: to gain insights on South American drivers’ perception of road safety and to provide scientific support to road safety policies by generating comparable national statistics on the present perception of road safety.

## 2. Materials and Methods

**Figure 1:**
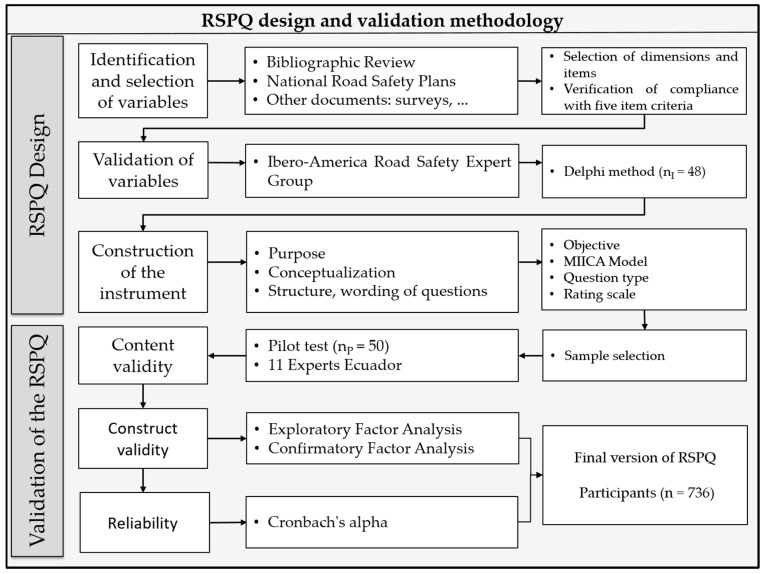
Synthesis of the methodology for RSPQ design and validation.

The development and validation of the Road Safety Perception Questionnaire (RPSQ) were performed in two main stages (design and validation), comprising six sub-stages, Figure 1 indicates the methodological process followed. The process for the construction and validation of the RSPQ is described below.

### 2.1. Design of the RSPQ (Development of a Draft Questionnaire)

#### 2.1.1. Identifying and Selecting RSPQ Variables

The identification and selection of variables in the RSPQ were carried out in two stages, as described below:

(a) Stage I: Identification and selection of road safety dimensions and items

Step 1: A literature review was carried out from 2010 onwards in different data sources (Red de Revistas Científicas de América Latina y El Caribe, ScienceDirect, Scopus, Google Scholar, and Scielo), using keywords such as road safety in Latin America, questionnaire validation, driver behavior, to identify previously validated questionnaires that assess the perception of road safety in the region for the following factors: human, vehicle, road infrastructure, regulatory framework, and regulatory and socio-economic measures.

Step 2: Nine National Road Safety Plans of South American countries were analyzed [45,46,47,48,49,50,51,52,53] to identify the items used in road safety, also, research documents developed by international organizations were included: Economic Commission for Latin America and the Caribbean [54], Traffic Safety Index [55]. The reports produced by the Observatorio Seguridad Vial en Latinoamérica (OISEVI), provide common indicators for the region [56], the Spanish Road Association, and the Inter-American Development Bank carried out several diagnostic studies on road safety in Latin American countries, in which common indicators and dimensions used in road safety were identified [57], other self-reported road safety questionnaires TSCI [35], and E-Survey of Road users’ Attitudes [39] were also used. The grouping of the items according to the selected dimensions was done using the conceptual framework of work by Aparicio [44], as described in Section 2.1.3 (section b).

(b) Stage II: Verification of compliance with RSPQ item criteria

The items identified in stage I were subjected to a validation process of compliance with five criteria, namely: relevant, measurable, understandable, non-redundant, and comparable, all extracted from the scientific literature [58,59]. The qualification process was carried out by four members of the group of researchers belonging to the Instituto Universitario de Investigación del Automóvil Francisco Aparicio Izquierdo of the Universidad Politécnica Madrid (INSIA-UPM), which consisted of verifying compliance (yes or no). This evaluation resulted in a reduction of the items to be submitted for validation by a group of road safety experts.

#### 2.1.2. Validation of the System of Variables to Make Up the RSPQ

To validate whether the variables are suitable for inclusion in the RSPQ, a group of Ibero-America experts was used applying the Delphi method, which is considered the most appropriate to achieve convergence of the responses of the road safety experts according to the literature consulted [60,61,62,63], and also this methodology is used when the information is insufficient or nonexistent [64], as in our case. Once the RSPQ items and dimensions were identified, the invitation to participate and the access link to the survey were sent by e-mail to the 109 road safety experts in Ibero-America. The consultation was conducted online using the SurveyGizmo tool and was carried out from September to December 2018.

The experts who responded to the survey were selected based on their “Expert Competence Coefficient”, with values between 0.8 and 1 being accepted as excellent, as recommended in the work of Zartha-Sossa [60]. The calculation of the “Expert Competence Coefficient” is based on a self-assessment of the expert’s opinion of his or her level of knowledge of the research problem, as well as the sources that allow him or her to argue the established criterion, for more details see [60].

#### 2.1.3. Construction of the RSPQ

The criteria for the construction of the RSPQ are (a) Purpose of the instrument, (b) Conceptualization of the instrument, (c) Structuring, drafting of the questions and instructions, following the works [65,66], which are summarized below.

(a) Purpose of the instrument

The purpose of the RSPQ was to analyze drivers’ perceptions as to the components of behavior, traffic regulations, infrastructures, vehicle safety and socio-economic aspects within the framework of road safety.

(b) Instrument conceptualization

The RSPQ design is structured in five dimensions (factors): (a) Human (FH), (b) Vehicle (FV), (c) Road infrastructure (FIV), (d) Regulatory framework and intervention measures (FN) and (e) Socio-economic (FS), according to the conceptual framework of the integrated research model proposed by Aparicio [44]. In addition to these dimensions, demographic aspects such as age, sex, city, gender, level of education, and type of driving license are included.

(c) Structure and composition of questions, presentation and instructions

The RSPQ used a matrix item structure and the response options a five-point Likert scale [67,68], with a rating of: 1 = very low, 2 = low, 3 = medium, 4 = high and 5 = very high. The questionnaires were applied individually and anonymously by previously trained surveyors. The questions were worded in common language to be understood by all the surveyed drivers. The instructions were read out and any doubts were clarified. The confidentiality and sincerity of the answers was assured to maximize the validity of the information obtained.

The interviewers were trained by the research team and had to meet a certain selection profile: they had to be university students, understand the research objective and know each question. For the fieldwork, they were instructed not to guide the interviewee during the survey and to limit themselves to capturing the information. For the selection of respondents, they were instructed to be gender-equitable and to comply with the percentages of the stratified sample. The main locations for the survey were bus stops, petrol stations, bus terminals, and Vehicle Technical Inspection Centers.

The survey was completed by the drivers themselves and they were informed about confidentiality and the importance of honest answers to maximize the validity of the information obtained. Only if they did not understand the question did the interviewer explain. However, some respondents did not complete the survey, and to avoid bias as much as possible, two additional filters were applied based on the time spent completing the questionnaire and the quality of the ratings. For example, given the 64-item questionnaire, respondents who completed the entire survey very quickly in e.g., less than 8 min were eliminated and, for items measured by the 5-point Likert scale, if the respondent rated all items with the same score, the response was also considered invalid.

The questions of each dimension are created with a common and manageable level of language for each driver to understand and be able to fill in. It was noted that incorrectly completed surveys are replaced, so 736 questionnaires were finally considered as valid responses from the Ecuadorian drivers to perform the analysis of the construct validity (Exploratory Factorial Analysis and Confirmatory Factorial Analysis) and evaluate the reliability of the RSPQ.

### 2.2. Questionnaire Validity

Before applying the RSPQ, a content validation process should be carried out, as previous studies have done [68,69,70]. For content evaluation, a pilot sample of 50 drivers and 11 Ecuadorian experts is used, as recommended by Hurtado and Tangarife [65,71] The construct validity is performed through Exploratory Factor Analysis and Confirmatory Factor Analysis and finally, the Reliability is performed with Cronbach’s Alpha but with a sample of 736 drivers. The process of validating the questionnaire is described below.

#### 2.2.1. Evaluation of RSPQ Contents

A pilot test of the RSPQ draft was carried out to resolve possible concerns or doubts about the wording or clarity of the questions. The pilot test was applied to a sample of 50 drivers as recommended [72,73]. This procedure collected feedback that helped define the draft RSPQ to be applied to a larger sample of drivers in Ecuador.

The 50 drivers who agreed to complete the instrument after being informed about the purpose of the study were surveyed under the following inclusion criteria: participants aged between 18 and 65 years, drivers of vehicles, motorcyclists, cyclists, and pedestrians, in possession of a valid driver’s license. Also, equality between male and female participants and a professional and non-professional license was considered, and divided by age strata: (a) 18 to 25, (b) 26 to 35, (c) 36 to 45, (d) 46 to 55 and (e) 56 to 65, with the number of respondents being: 11, 13, 11, 8 and 7, respectively.

After making the corrections suggested by the 50 drivers to the RSPQ, it was evaluated by eleven Ecuadorian experts following the recommendations of Jiménez [74] and Hurtado [65], who evaluated the appropriateness, importance, and clarity of the RSPQ content, through a questionnaire according to Guevara’s suggestion [75]. The appropriateness and importance are evaluated through a 5-point Likert scale (1 = very low and 5 = very high) and clarity (dichotomous yes/no) [71,76]. The criteria for eliminating items questionnaire is valuing below 3, modifying items with values between 3.1 and 3.5, and accepting items above 3.6 [77].

The eleven Ecuadorian experts who had a university degree and more than eight years of experience in road safety evaluated the RSPQ.

#### 2.2.2. Construct Validity

The RSPQ construct was validated by an Exploratory Factor Analysis (EFA) and Confirmatory Factor Analysis (CFA) following previous similar studies [68,70]. The EFA was carried out using the SPSS 25 software [78] and reported Kaiser-Meyer-Olkin values for the suitability of the sampling higher than 0.60 (the closer to 1 the better), also the Bartlett sphericity test (significant at 0.05) for the established dimensions, and the variance explanation level with a value equal to or higher than 50% for the dimensions. The number of initial factors was identified by a sedimentation graph, whose components must be over a self-value. To identify the factorial loads we used the Varimax Orthogonal Rotation with Kaiser normalization, in which each factor is independent of the others and finds an adequate correlation between the variables observed by the factor. The factorial loads are expected to be equal to or higher than 0.50.

The CFA was performed with the AMOS 24 software [79] and evaluated multivariate normality by means of the Mardia Test. After eliminating outliers by means of Mahalanobis distances the sample was found not to violate this principle. As recommended in [79] a bootstrapping of 500 samples was performed by the Bias-corrected percentile, and according to the 95% intervals all the items and factors made a significant contribution to the weight of the regressions and intercorrelations, respectively.

It was then decided to use the Maximum Verosimilarity method to analyze the dimensionality, adopting the fit indices recommended by Hair et al. [80] and [79]. Those considered were the minimum discrepancy (CMIN), most commonly expressed as a χ2 statistic, which due to the size of the sample was expected to obtain a significant p value; Goodness-of-Fit Index (GFI) which should have a value close to 1 without established thresholds; Mean Square Residual (RMR) which should be around ≤0.08 as long as the CFI is >0.90; Root Mean Square Error of Approximation (RMSEA), which should not be less than 0.07 when the CFI is >0.90.

The incremental adjustments considered the Adjusted Goodness-of-Fit Index (AGFI), which should be over 0.900, normal values are around 21 without thresholds; Comparative Fit Index (CFI), Incremental Index of Fit (IFI) and Tucker-Lewis Index (TLI), whose thresholds should be higher than 0.90; Normalized Fit Index (NFI) which should be over 0.95 or 0.90 but this can be replaced by a good CFI adjustment. The CMIN was used as the Parsimony Index which is equivalent to the CMIN/DF, whose values should be between 2 and 3.

#### 2.2.3. Reliability

To assess the reliability of the questionnaire, Cronbach’s alpha [81], was used, evaluating only in terms of internal consistency as suggested by [65,67], applying the item covariance method of the dimensions applied to the Likert scale questions from the RSPQ data applied to 736 drivers. After showing the validity of the questionnaire, its reliability was also shown by Cronbach’s alpha [82], for which 0.80 is considered a good score and 0.90 is excellent [83].

### 2.3. Sample Selection

A Proportionate Stratified Random Sampling method was used in five age categories between 18 and 65 years of age, both in a pilot sample and in the application of the questionnaire, according to the population census of Ecuador of the Ecuadorian Institute of Statistics and Census [84].

The completion of the questionnaire was carried out in a definitive sample based on the estimated number of drivers in Ecuador [85] of 4492, 929 according to 2017 data, with a confidence level of 97%, 4% error, and 50% heterogeneity, also, equality was sought between participants of male and female gender and professional and non-professional license, divided into five age strata, taking into account the percentage of Ecuadorian population distribution: (a) 18 to 25, (b) 26 to 35, (c) 36 to 45, (d) 46 to 55 and (e) 56 to 65, being the number of surveys to be conducted of: 166 (23%), 192 (26%), 163 (22%), 124 (17%) and 91 (12%) respectively which resulted in a recommended sample of 736 drivers. This sample will be used for construct validity and internal reliability of the instrument.

The RSPQ was applied in the three main cities of Ecuador (Cuenca, Quito, and Guayaquil), which were chosen because they have the largest number of inhabitants and the highest number of traffic accidents [5]. The number of surveys applied in the three cities is balanced. In those cases, where questionnaires were identified as incorrectly filled out (generating atypical data) or incomplete, the questionnaires were replaced [84]. Table 1 presents some data for the three selected cities and the national total.

In Ecuador, 49.6% are men and 50.4% are women. Quito and Cuenca (highlands) are the most socially and economically developed cities when compared internally with Guayaquil, which belongs to the coastal region [86].

### 2.4. Final Version of Questionnaire

From the results of the previous steps identified, we created the final questionnaire that was used for the study. This work shows the construction process of the instrument applied to the sample of 736 Ecuadorian drivers, for construct validity and reliability.

## 3. RSPQ Survey Participants: Descriptive Data

The field study was conducted with 736 Ecuadorian drivers. The socio-demographic characteristics of the respondents are shown in Table 2.

It is a sample concentrated in the 18–35 age range (49%), with only 12% of respondents being over 55 years old (of the 736 respondents, the average age is 37.53 years). It is an educated population, with levels of education falling into two main categories: “completed secondary education” (36%) and “completed university education” (51%). The sample is predominantly male (71%) than female (29%) and more than half have a non-professional driver’s license (60%).

## 4. RSPQ Design and Validation Process Results

### 4.1. Identification and Selection of RSPQ Variables

Unfortunately, the review of the scientific literature did not yield good results, the number of works found was very small and those that were identified are not directly related to the subject under investigation. From the National Road Safety Plans and international organizations and OISEVI, a set of 184 variables were initially identified, a figure that was considered very high and not very applicable, so a significant reduction of the items was made to a total of 93, keeping only the common indicators at the beginning and those that could provide substantial information on road safety in South American countries, such as speeding, driving after drinking alcohol. The 83 items were checked for compliance with five criteria (relevant, measurable, understandable, non-redundant, and comparable) of the RSPQ items, resulting in 74 items.

### 4.2. Validation of Questionnaire Items

The 74 items initially obtained from the literature review and other sources of information were evaluated by Ibero-American road safety experts using the Delphi technique [60], the composition of the group of experts was established according to the fulfillment of a professional profile, e.g., Transport and Road Engineering, In this sense, a database of 109 Iberoamerican road safety experts was obtained, obtaining a response rate of 69% [75], from which the responses of the 48 Ibero-American road safety experts were selected according to their “Expert Competence Coefficient”, which is between the range of 0.8 and 1 as excellent as recommended in the work of Zartha-Sossa [60]. This selected group had an average of 14 years of experience and represented 11 Ibero-American countries (Argentina 29%, Bolivia 4%, Colombia 6%, Ecuador 21%, Spain 9%, Chile 8%, Costa Rica 2%, Mexico 4%, Peru 11%, Uruguay 4%, and Venezuela 2%). Once it was verified that the experts had the necessary competencies, the responses to the assessment of the road safety items are presented, most of which suggested eliminating redundant items and clarifying the wording to facilitate the driver’s understanding. Based on these suggestions, the instrument was composed of a total of 64 items and six socio-demographic data, see Table 3.

#### Evaluation of Contents

The results of the evaluation made by the 50 drivers to the RSPQ were very general related to the comprehension of a few terms such as the following:

- In the sociodemographic data section, it was detected that the phrase “year of birth” generated problems when tabulating the answers so it was replaced by “age”.
- It was detected that the phrase “Validated license” was difficult to interpret in more than 40% of the cases, so it was decided to replace it with the term “expired license”.
- The phrase “traffic circle or traffic circle” was found to be difficult to interpret in more than 70% of the cases, so it was replaced by the term “traffic circle”.
- The wording of items FH8, FV7, FIV12, FIV13, and FN6 was adjusted.

Besides, the RSPQ did not incorporate new items or eliminate items from the questionnaire and did not present problems with the internal order of the survey. The questionnaire proposed by Guevara [75] was used to evaluate the RSPQ contents. Items that scored below 3 were eliminated, those between 3.1 and 3.5 were modified, and those above 3.6 were accepted [77]. As the experts scored the dimensions with an average value of more than 4, we ratified the validity of the contents initially proposed by the international panel of South American experts. None of the dimensions scored less than 3 and most were over 4. For this reason, the ratings issued by them do not have values lower than three and most of them are higher than four.

### 4.3. Validity of Construct

#### 4.3.1. Exploratory Factor Analysis (EFA)

As the original RSPQ of 64 items and five road safety dimensions presented unsatisfactory results on the level of variance explained, it was combined with the Confirmatory Factor Analysis (CFA) to identify problematic items. When the items had been reduced the EFA was re-run and the results showed that the Kaiser-Meyer-Olkin measure of 0.952 possessed a good quantity of data for this process. Also, Bartlett’s Sphericity Test (approximately Chi-square 14,272.22 (82 gl); sig. = 0.000) showed that the factorial analysis was appropriate. The number of items to be extracted was identified by the sedimentation graph, which had an elbow above the eigenvalue of the six dimensions, which were assessed by the accumulated proportion of variance of 55.56% of explained variance and was considered acceptable.

On reviewing the factorial loads that best defined a factor it was seen that there were now seven factors instead of the original six. The component matrix was rotated to find a simple solution. The Varimax orthogonal rotation generated an average of minimal factorial loads of 0.514 and an average maximum of 0.712. Each of the six dimensions the factorial load of the legal framework and regulatory framework and intervention measures (FN) was between 0.323 and 0.722; the road infrastructure factor (FIV) was between 0.590 and 0.698; the vehicle factor (FV) was from 0.488 to 0.638; and the socio-economic factor (FS) from 0.597 to 0.769.

The human factor presented a division into two parts, one for general driving conduct, which retained the name of human factor (HF) with a factorial load of 0.401 to 0.763; while the human factor also contained items mainly pertaining to driving precautions (FPC) with loads from 0.625 to 0.681. The FN had the lowest factorial load and FS the highest. All the dimensions had acceptable factorial loads for the model, although item FN16 had the lowest load, see Table 4.

#### 4.3.2. Confirmatory Factor Analysis

Four models were reported; the first two were original theoretical models while the others were similar to the EFA (see Table 5). Model 0 was the original model proposed with 64 items grouped into five factors, although the absolute adjustment index (GFI) and incremental indices presented inadequate fits. In the subsequent models the covariance errors were corrected in each factor and items were eliminated with very low factorial loads or excessive number of covariance errors or high standardized residuals of covariance. Model 1 considered all 64 items and five factors. Model 2 also considered five factors although it created two endogenous variables from the dimensionality suggest by the EFA. Model 3 identified the endogenous dimensionality of the variables and considered them as factors due to the FPC semantics pointing out human factors in relation to vehicle dynamics and driving care.

On comparing the indices of the three models (see Table 5) Models 2 and 3 were found to present the best fits, although the most similar to the ideal model was Model 3, which complied with all the requisites proposed by Byrne and was close to the threshold of 0.90 suggested by Hair [80] for AGFI and NFI. The absolute indices complied with all the fits while the incremental indices were closest in Model 3.

Table 6 gives the standardized factorial loads. The lowest average of the six factors was 0.603 and the highest 0.741. FN had the lowest factorial load although the item with the least load was not in this factor but in el FH (0.529). The highest factor belonged to the socio-economic area, in spite of which the results presented very good standardized factors in all cases.

### 4.4. Reliability and Factorial Interrelationships

Table 7 gives the factorial intercorrelations with the reliability coefficient calculated by Cronbach’s alpha. There was a good general level of 0.950, while all the factors separately presented good reliability, with a coefficient of >0.80. [81,82,87]. The factorial intercorrelations had an average of >0.60. The lowest intercorrelations were found in FS in relation to HF (0.403) and FPC (0.425).

## 5. Discussion

The RSPQ arose from the need for a specially designed tool to determine drivers’ perception of road safety for use in future studies. In addition to the requirement of road safety researchers for instruments that are reliable and valid, and given that multiple measures are typically incorporated in programs of research, parsimonious tools are fundamental [88]. The behavioral data, besides that on accidents and infractions, can be used to measure the effectiveness of specific counter-measures applied to road safety, since each country has its own problems in relation to drivers’ behavior [89].

Therefore, the main objective of this study was to design and describe in detail the validation of the self-reported comprehensive questionnaire of drivers’ perception of road safety. Initially, the theoretical survey consisted of five factors, which in the course of the EFA and CFA were modified to six factors (vehicle (FV), road infrastructure (FIV), regulatory framework and intervention measures (FN), socio-economic (FS), human (FH), and the new one defined as *driving precautions* (FPC). Theoretically, the 64-item version of the RSPQ is relevant, however, only the 41 items are validated on a six-factor or six-dimension structure.

The results of the six-factor model showed that the RPSQ fitted in satisfactorily with this structure. These labels responded firstly to the most heavily weighted items in each factor, and secondly to the theoretical background of the “Development and application of an integrated methodology for the study of road accidents with involvement of vans” [44], the extraction of the items of the National Road Safety Plans of different South American countries [45,46,47,48,49,50,51,52,53], and from several technical reports on road safety in South America carried out by recognized international institutions [56] and the support of the available scientific literature [35,56,90].

The validity of the RSPQ constructs, the fit of model three comprising six factors (dimensions) with 41 items meets all the recommendations suggested by Byrne [79] and is close to reaching the thresholds suggested by Hair [80] for the incremental fits of the AGFI and the NFI. Ultimately, model three meets all the absolute index fits, while the incremental indexes are better approximated in the incremental indexes compared to the theoretical model (model 0) of five factors with 64 items. Only one goodness-of-fit index speaks against the fit of model three, with a p-value of less than 0.001 and the AGFI of 0.885, but the χ^2^ value (1594) was within the limits indicated by Wheaton et al. [91] and Carmines and MacIver [92] and the RMSEA (0.039), i.e., exceeded the criteria suggested by Browne and Cudeck [93]. In this sense, it was decided to eliminate items with a low factor loading of less than 0.40 (except for the item “Inadequate or poor training and monitoring of traffic control officers”, which was considered very important). This six-factor factor structure obtained a variance of 55.46%, which is considered acceptable [78].

The following items were eliminated from the human factor: FH2, FH5, FH6, FH8, FH9, FH13, and FH15; from the vehicle factor: FV1 and FV4; from the infrastructures factor: FIV1, FIV4, FIV7, FIV9, FIV11, and FIV12; from the legal and intervention measures factor: FN1, FN5, FN12, FN13, FN14 and FN15; and from the socio-economic factor: FS1 and FS7. The elimination of these items provided a parsimonious RPSQ and reduced the time required to complete the questionnaire. These items also had a low factorial load according to the EFA. Of the factors with the lowest factorial load, the legal framework and intervention measures (FN) had the most problems, so that future studies should pay attention to its dimensionality. All the factors have acceptable factorial loads for the model, although Item FN16 had the lowest, which is theoretically important since it supports the lack of drivers’ understanding of the vehicle’s dynamic behavior at excessive speeds and with excessive loads. This will allow driving schools to modify and reinforce the associated teaching material.

In fact, the CFA results revealed that the six-factor model was the best, indicating that the human factor, which originally had 19 items and was divided into two to form a new factor with five items, and confirmed the six factors structure. The questionnaires also support distinguishing between the drivers’ behavioral and drivers’ understanding of the vehicle’s operative conditions, denoted as *driving precautions* [94,95].

The Cronbach’s Alpha of the CFA for the six dimensions ranged between 0.823 and 0.885, which was over the minimum value of 0.70 for internal consistency [80] and with generally good reliability of 0.950 [87,96] Most of the dimensions’ intercorrelations were generally high, with the exception of the FH and FIV social factors, with a low correlation. This should be evaluated in other samples to verify whether the identity of the construct is affected by including or eliminating this dimension.

The lowest reported scores were within the legal framework and intervention measures factor, while the highest was obtained for the socio-economic construct.

One of this study’s strong points is the novel base of our questionnaire, which includes more road safety dimensions (socio-economic, legal framework) than the usual ones (human, vehicle, road infrastructure). The questionnaire can be self-administered and can also be implemented online, which is an advantage for large-scale applications [97,98] Some of its other strong points are the inclusion of a panel of 48 Ibero-American experts plus the 11 national RPSQ experts who validated the content of the questionnaire, following the recommendation of Hyrkäs [99], in which an item can be included in the instrument if 80% of the experts agree its validity, as was the case in our study.

The RSPQ survey differs in its construction process through the application of a methodology for the identification of the dimensions and corresponding indicators, taking into account the characteristics of the Latin American region. From the documents consulted (national road safety plans), which is also used for the construction of the Traffic Safety Culture Index [34], of the self-report instruments used in road safety in Latin America, there is no development of the construction process [39] and psychometric evidence of the instrument. Besides, some items do not discriminate between different types of drivers (professional and non-professional), an aspect that is relevant from the point of view of road safety [41].

The application of the RSPQ in cities in Ecuador aims to assess the behavior of drivers, the characteristics and level of presence of safety systems in the vehicle fleet, the level of knowledge and development of the regulatory framework and intervention measures, the state of the road network and the socio-economic level. The conceptualization of the survey includes road safety problems specific to regions and countries other than those in Europe [18]. This contribution aims to contribute valuable information for road safety in countries that do not have homogeneous, complete, and accessible accident information systems for studies and scientific research on traffic accidents, as is the case in Ecuador. Future research would also warrant the collection of information on the perceptions not only of drivers but also of other road users such as pedestrians and cyclists, which would contribute to assessing the usefulness and practicality of this tool on a broader level.

### 5.1. Limitations of the Study

Our results may not be fully representative of Ecuador since only the three cities with most traffic accidents were selected: Cuenca, Guayaquil and Quito, which limits its generalization. Other confidentiality and validity aspects will need to be examined for its application in other South American countries. The selection of the panel of safety experts may also have been biased as all the members were volunteers.

There are also certain limitations regarding the use of the 5-point Likert scale; the structural equations of Maximum Likelihood are known to function better with numerical than ordinal variables.

The study may be limited by the bias associated with the self-administration of the questionnaire, particularly its potential to obtain socially desirable responses [3]. However, it is important to point out the findings of Ajunen & Summala [100], who found that the influence of socially desirable responses was not substantial and especially when a questionnaire is completed in privacy. The participants in the present study filled out the questionnaire in their own time and were assured of confidentiality for their responses, so that the impact of social desirability can be expected to be minimal. However, since validation is a continuous process, further studies will be needed to determine the most effective form of using this tool to ratify its validity.

### 5.2. Prospects

The RPSQ can be applied to different fields as long as a validation of the instrument is carried out. The present study has filled in certain gaps in the literature as regards the lack of reliable and valid instruments for obtaining information on drivers’ perception of road safety in the developing countries due to the high South American mortality rate...

This questionnaire can be used to gain a better understanding of why the road safety strategies implemented by the National Safety Plans are not managing to reduce road deaths by 50% [1], as expected, and as the first step in reforming road safety strategies. The analysis of the RSPQ responses can indicate the appropriate steps in this direction to obtain more responsible and safer driving habits and help to establish better safety strategies to reduce the number of accidents and deaths on the roads.

Future studies will of the RSPQ tool be expanded by other road user groups (by type of vehicle, motorcycles, pedestrians, bicycles), estimation of the number of kilometers traveled annually, to assess the risk of having suffered traffic accidents in recent years, and to go deeper into aspects such as speed, alcohol, driving styles, among others.

## 6. Conclusions

In conclusion, this RPSQ has shown the validity of its preliminary contents. The confirming Factorial Analysis and the Exploratory Factor Analysis, of the integral questionnaire on the perception of road safety have the optimal structure of 41 items grouped into six dimensions, although five dimensions could also have been used with an endogenous variable to sub-divide the human factor into driving behaviors and precautions. From the fit indices of the confirmatory analysis, it can be concluded that the scale has adequate dimensionality, while its reliability coefficient ensures that the instrument’s items fulfill their function of assessing drivers’ perceptions of the respective dimensions of road safety. The methodology used to create and validate the questionnaire, with a panel of experts, is a good fit.

The RSPQ could contribute to the understanding of how drivers perceive road safety in a region, as well as to improve the articulation of the implementation of road safety strategies. This study fills a gap in the existing literature related to the lack of reliable and valid instruments available in South America to collect information on perceptions of road safety factors, regulatory framework and intervention measures, road infrastructure, vehicles, socioeconomic, human, and driving precautions.

## Figures and Tables

**Table 1:** Social and accident characteristics in 2017 for the three provinces of Ecuador.

City	Total Inhabitants	Number of Traffic Accidents (TA)	Number of Fatalities in TA	Number Injured in TA	Total Vehicles
Quito	2,644,145	9363	375	5345	511,782
Guayaquil	2,644,891	8422	456	8081	480,977
Cuenca	603,269	1497	92	1127	141,848
Total at national level	167,769,77	28,967	2153	22,018	2,237,264

**Table 2:** Socio-demographic characteristics of the 736 drivers with valid questionnaires.

Variable	Group	Frequency	Percentage (%)
Gender	Male	522	70.92
	Female	214	29.08
Age Group	18–25	166	23
	26–35	192	26
	36–45	163	22
	46–55	124	17
	55–65	91	12
Driving license	Professional	294	39.95
	Non-professional	442	60.05
Education Level	Primary School	72	9.78
	High school—Secondary studies	265	36.01
	University studies	376	51.08
	University studies—Postgraduate	23	3.13

**Table 3:** Questionnaire and construct descriptions.

Human Factor	
Evaluate the influence of the following aspects of driving behavior according to your criteria on a large number of road accidents and victims in Ecuador.	
**Items**	
FH1	Driving without using and not requiring all occupants of the vehicle to wear seat belts
FH2	Driving without using a child car seat (child seat)
FH3	Motorbike riders driving without a helmet
FH4	Driving at speeds over the established legal limit
FH5	Driving the vehicle at excessive speed in adverse road weather conditions (rain, strong wind, poor visibility, etc.).
FH6	Using a mobile phone while driving
FH7	Sending messages while driving
FH8	Driving without a valid license or with an expired license
FH9	Driving under the influence of drugs, medicines, or other inhibitory substances
FH10	Driving with high levels of fatigue or tiredness (sleepiness)
FH11	Driving after the consumption of alcohol beyond the legal limits
FH12	Driving without respecting vertical signs: stop, give way, speed limit, etc.
FH13	Driving without respecting horizontal signs: turns, white lines on the pavement, etc
FH14	Driving with excess weight inside the vehicle
FH15	Driving with an excess load on the outside of the vehicle
FH16	Driving with more than the permitted number of passengers
FH17	Driving without reflective materials, especially cyclists
FH18	Driving with one of the vehicle’s headlights burnt out
FH19	Driving without keeping a safe distance from other vehicles
**Vehicle factor** According to your criteria, evaluate the influence of possible safety deficiencies of the present Ecuadorian vehicle fleet in the high number of traffic accidents and victims.	
**Items**	
FV1	Not complying with the regulations regarding vehicles
FV2	The high proportion of vehicles in the fleet without seat belts
FV3	The high proportion of vehicles in the vehicle fleet that does not have anti-lock braking systems (ABS).
FV4	The high proportion of vehicles in the fleet that does not have Electronic Stability Control (ESP)
FV5	The high proportion of vehicles in the fleet that does not have driver and passenger frontal airbags
FV6	The high proportion of vehicles in the vehicle fleet does not have a speed limiter (heavy duty vehicle)
FV7	The high proportion of vehicles without tachographs (heavy vehicles, record of events while driving)
FV8	The high proportion of vehicles without technical inspection certificates
FV9	The high average age of the vehicle fleet.
**Road infrastructure factor** According to your criteria, evaluate the influence of possible deficiencies of Ecuadorian roads on the high number of accidents and victims.	
**Items**	
FIV1	Shortage of vertical signals or inadequate or badly maintained signals (stop, give way, etc.)
FIV2	Inadequate or badly maintained horizontal signals (white lines on the road, arrows, etc.)
FIV3	Poor road maintenance
FIV4	Low investment programs dedicated to road infrastructure safety
FIV5	Dangerous bends
FIV6	Side barriers: scarce, inadequate, or inadequately maintained
FIV7	Shortage or inadequate design of road shoulders, or sidewalks (shoulder, roadside on either side of the road).
FIV8	Lighting deficiencies on interurban roads (Highway)
FIV9	The pavement of the main roads in a poor state of conservation
FIV10	Road pavement of secondary roads in a poor state of preservation
FIV11	The low proportion of high-capacity road network (road)
FIV12	The low proportion of high-capacity network (highways, or similar)
FIV13	The low proportion of high capacity network (motorways, or similar)
**Regulatory framework and intervention measures factor.** According to your criteria, evaluate the influence of possible deficiencies of the present regulations and intervention measures in the number of road accidents and victims in Ecuador in the following aspects.	
**Items**	
FN1	Low compliance with the regulations on road safety
FN2	Low training requirements for driver licensing in general
FN3	Inadequate or scarce training for professional drivers
FN4	Low effectiveness of the sanction system: lack of enforcement
FN5	The low standards required by the regulation and approval of vehicles
FN6	Small number of alcohol tests applied
FN7	Small number of speed controls
FN8	Small number of controls on using phones or other devices while driving
FN9	Small number of controls for helmets by motor bike riders
FN10	The low level of compliance with rules on consumption of alcohol while driving
FN11	Scarce presence of police on highways
FN12	Few communication large campaigns in the press radio and TV
FN13	Small number of restricted speed zones in cities
FN14	Scarce of inadequate signaling of accident black spots on the roads
FN15	Regulations on the low standards required in technical vehicle inspections
FN16	Inadequate or inadequate training and control of traffic officers
**Socio-Economic factor** According to your criteria, evaluate the influence of possible deficiencies in the following socio-economic aspects on the high number of traffic accidents and victims in Ecuador.	
**Items**	
FS1	Low economic level of many citizens and its influence on mobility, and technological level of vehicles
FS2	Inadequate attitudes of citizens regarding compliance with traffic regulations
FS3	Low level of general information of citizens concerning traffic, road safety, and the impacts of traffic accidents
FS4	Low-level training of citizens concerning traffic, road safety, and the impacts of road accidents
FS5	Inadequate perception of citizens concerning the main risk factors and their influence on road accidents and the number of victims: speed, alcohol and drugs, use of means of protection, and others
FS6	Low level of development of social movements and associations of road accident victims (foundations, road safety observatories).
FS7	Low media coverage of road safety issues and road traffic accidents

**Table 4:** Factorial loads by the principal components extraction method and Varimax rotation with Kaiser normalization.

Items	Components					
	FN	FH	FIV	FPC	FV	FS
FN8	0.722					
FN9	0.716					
FN7	0.675					
FN6	0.634					
FN10	0.629					
FN11	0.570					
FN4	0.558					
FN2	0.496					
FN3	0.459					
FN16	0.323					
FH11		0.763				
FH4		0.733				
FH3		0.678				
FH10		0.666				
FH7		0.621				
FH12		0.573				
FH1		0.401				
FIV6			0.698			
FIV5			0.679			
FIV3			0.664			
FIV13			0.624			
FIV10			0.621			
FIV8			0.609			
FIV2			0.590			
FH16				0.681		
FH14				0.678		
FH19				0.678		
FH17				0.652		
FH18				0.625		
FV6					0.638	
FV7					0.637	
FV5					0.623	
FV9					0.565	
FV8					0.554	
FV3					0.543	
FV2					0.488	
FS3						0.769
FS4						0.760
FS5						0.665
FS2						0.646
FS6						0.597

**Table 5:** Absolute, incremental, and parsimony indexes for the known models.

Models	χ2	Absolute Indices			Incremental Indices					Parsimony Indices	
		GFI	RMSEA	RMR	AGFI	TLI	CFI	NFI	IFI	CMIN/DF	AIC
0	5974.558 *	0.758	0.054	0.052	0.739	0.834	0.841	0.783	0.842	3105	6286.558
1	1795.625 *	0.882	0.044	0.040	0.864	0.916	0.924	0.877	0.924	2404	2023.625
2	1608.139 *	0.900	0.040	0.038	0.885	0.931	0.937	0.889	0.938	2150	1834.139
3	1594.566 *	0.901	0.039	0.037	0.885	0.932	0.938	0.890	0.938	2140	1826.566

Note: * *p* < 0.05; GFI = goodness of fit index; RMSEA = root mean square error of approximation; AGFI = Adjusted Goodness-of-Fit Index; TLI = Tucker-Lewis index; CFI = comparative fit index; NFI = Normed Fit Index; IFI = Incremental Index of Fit; CMIN/DF = chi-square divided by degrees of freedom; AIC = Akaike information.

**Table 6:** Standardized Regression Weights from Confirmatory Factor Analysis.

Items	Component					
	FH	FV	FIV	FN	FPC	FS
FH4	0.659					
FH3	0.660					
FH1	0.529					
FH7	0.624					
FH10	0.729					
FH11	0.680					
FH12	0.710					
FV2		0.619				
FV3		0.642				
FV5		0.706				
FV6		0.742				
FV7		0.714				
FV8		0.622				
FV9		0.594				
FIV2			0.636			
FIV3			0.631			
FIV5			0.698			
FIV6			0.688			
FIV8			0.617			
FIV10			0.698			
FIV13			0.629			
FN2				0.584		
FN3				0.600		
FN4				0.631		
FN6				0.674		
FN7				0.673		
FN8				0.634		
FN9				0.680		
FN10				0.657		
FN11				0.654		
FN16				0.640		
FH14					0.658	
FH16					0.690	
FH17					0.790	
FH18					0.763	
FH19					0.711	
FS5						0.638
FS4						0.742
FS3						0.804
FS2						0.685
FS6						0.698

Note: A bootstrapping of 500 samples was added by the Bias-corrected percentile method according to the 95% intervals. All the items were found to make a significant contribution to Model 3.

**Table 7:** Factorial intercorrelations and reliability coefficients (Cronbach’s Alpha).

Factor	FPC	FH	FV	FIV	FN	FS
FPC	(0.851)					
FH	0.714	(0.852)				
FV	0.610	0.698	(0.848)			
FIV	0.624	0.647	0.709	(0.848)		
FN	0.707	0.680	0.747	0.721	(0.885)	
FS	0.425	0.403	0.633	0.500	0.630	(0.823)

Note: A bootstrapping of 500 samples was added by the Bias-corrected percentile method according to the 95% intervals. All the factors were found to have a significant correlation.

## Data Availability

Not applicable.
